# Research on Electromagnetic and Rheological Performance of Microwave-Sensitive Emulsified Asphalt Containing SiC and Fe_3_O_4_

**DOI:** 10.3390/ma18184283

**Published:** 2025-09-12

**Authors:** Peng Wu, Shuyin Li, Haoyan Guo, Haibao Zhang, Rui He

**Affiliations:** 1 Infrastructure Department, Chang’an University, Xi’an 710061, China; 2 School of Materials Science and Engineering, Chang’an University, Xi’an 710061, China; 3 Shaanxi Union Research Center of University and Enterprise for Advanced Transportation Infrastructure Materials, Xi’an 710061, China

**Keywords:** emulsified asphalt, electromagnetic properties, microwave heating efficiency, rheological properties, SiC, Fe_3_O_4_

## Abstract

The limited microwave-heating performance caused by moisture and ordinary aggregates limits the application efficiency of emulsified asphalt in rapid pavement repair engineering. Silicon carbide (SiC) and ferrosoferric oxide (Fe_3_O_4_) were introduced as modifiers to prepare the microwave-sensitive emulsified asphalt used in this work. The electromagnetic properties, microwave heating properties, microstructural evolution law, and rheological performance of emulsified asphalt or its evaporation residue were studied. The results show that modification through SiC and Fe_3_O_4_ can produce a pronounced synergistic effect and can significantly enhance both the electromagnetic and high temperature rheological properties. Coupling polarization enhancement with magnetic responsiveness increases the dielectric constant and loss peaks compared with single doped samples. This compensates for the weak magnetic response or insufficient stiffness of single doped systems and leads to a maximum early-stage microwave heating rate increase of 176.2%. The rheological performance of the compound doped system is also markedly improved. The R (3.2 kPa) of the 2% SiC + 3% Fe_3_O_4_ group sample increased by 59.7% and the *J_nr_* (3.2 kPa) decreased by 68.9% compared to the control group. The rigid and elastic complementarity of the two modifiers effectively suppresses irreversible deformation at high temperatures. Moreover, the modifiers accelerate the microstructural transition of the asphalt from a particulate state to a continuous phase under microwave exposure. Adjusting the compound doping ratio of SiC and Fe_3_O_4_ allows the system to be tailored for either high temperature stability or rapid heating, providing technical support for its application in microwave-assisted pavement repair field.

## 1. Introduction

Microwave-sensitive emulsified asphalt, as a key material for microwave-assisted pavement repair technology, can rapidly absorb microwave energy and convert it into thermal energy. This property significantly shortens pavement maintenance periods and enhances repair efficiency, making it highly valuable in emergency road repair and rapid maintenance applications [1,2]. In contrast, conventional emulsified asphalt exhibits weak electromagnetic responsiveness (the real part of complex permittivity *ε*′ is only 2.1–2.3, and real part of complex permeability *μ*′ is close to 1 in the 8.2–12.4 GHz frequency range) and low energy absorption efficiency (the early-stage microwave heating rate is merely 0.8 °C/s within 0–60 s) in microwave fields, which makes it difficult to meet the technical requirements of rapid heating and uniform warming in microwave repair processes [3,4]. Microwave-sensitive emulsified asphalt modified with dielectric materials such as SiC, TiO_2_, and Al_2_O_3_ is endowed with superior microwave sensitivity, effectively addressing this limitation and emerging as a research hotspot in the field of pavement materials in recent years [5].

The core advantage of microwave-sensitive emulsified asphalt lies in its ability to efficiently absorb and convert microwave energy into other types of energy, which primarily depends on the selection and compounding design of modifiers [5,6]. Currently, commonly used microwave-sensitive modifiers are mostly of a single type, such as the dielectric-type (e.g., carbon materials, ceramic particles) or the magnetic-loss-type (e.g., ferromagnetic metal oxides) [7,8,9]. Although single dielectric-type modifiers can enhance dielectric loss, insufficient magnetic responsiveness results in a relatively narrow microwave absorption band. Conversely, single magnetic-loss-type modifiers can improve magnetic loss but suffer from limited structural stability at high temperatures. Therefore, neither type of modifier can achieve the synergistic optimization of microwave sensitivity and high temperature stability individually [10,11,12].

High temperature rheological stability is a critical prerequisite for the engineering application of microwave-sensitive emulsified asphalt. It determines the rutting resistance of repaired pavements under hot summer conditions and their long-term service life [13,14]. Microwave-sensitive emulsified asphalt must withstand repeated vehicular loading after microwave heating and curing. If the material exhibits a low complex modulus (less than 1.5 MPa at 60 °C, a typical high temperature for pavement in China) and weak elastic recovery capacity at elevated temperatures, permanent deformation tends to accumulate, leading to pavement distresses such as rutting and shoving [15,16]. Therefore, when preparing microwave-sensitive emulsified asphalt, it is essential to simultaneously ensure both high microwave sensitivity and excellent high temperature rheological properties. Focusing only on microwave heating efficiency may compromise high-temperature properties. The combined incorporation of silicon carbide (SiC) and ferrosoferric oxide (Fe_3_O_4_) offers a promising strategy for achieving the synergistic enhancement of high microwave sensitivity and superior high-temperature stability. SiC possesses outstanding dielectric properties and high hardness. It not only enhances microwave absorption through dielectric loss but also provides a rigid skeleton to the asphalt matrix, thereby improving resistance to deformation at high temperatures [17,18]. Fe_3_O_4_, on the other hand, exhibits strong magnetic responsiveness, which strengthens magnetic loss via magnetic resonance and eddy current effects, broadens the microwave absorption band, and improves the elastic recovery capacity of asphalt [19,20]. Their combined use is expected to enhance microwave sensitivity through dielectric–magnetic loss synergy while optimizing high-temperature performance via rigid–elastic complementarity. However, the regulatory mechanisms and underlying principles governing this composite modification system for microwave-sensitive emulsified asphalt remain to be further explored.

Therefore, this work focuses on the preparation and performance evaluation of microwave-sensitive emulsified asphalt, with SiC and Fe_3_O_4_ employed as composite modifiers. Different single doping and compound doping modification schemes with varying proportions were designed to prepare microwave-sensitive emulsified asphalt. The microwave sensitivity and service performance of the modified asphalt were systematically tested. Microstructural observations were conducted to reveal the synergistic mechanism of SiC and Fe_3_O_4_. This work aims to provide theoretical guidance and technical support for the development and engineering application of high-performance microwave-sensitive emulsified asphalt.

## 2. Experiments

### 2.1. Materials

SiC and Fe_3_O_4_ were selected as electromagnetic modifiers in this work. The chemical composition of SiC is presented in Table 1. Its physical properties are listed in Table 2. The Mohs hardness scale is used to measure the scratch resistance of minerals, with 1 representing the softest and 10 representing the hardest. A Mohs hardness of 9.3 for SiC indicates that it has excellent scratch resistance and can provide a rigid skeleton for the asphalt matrix.

**Figure 1:**
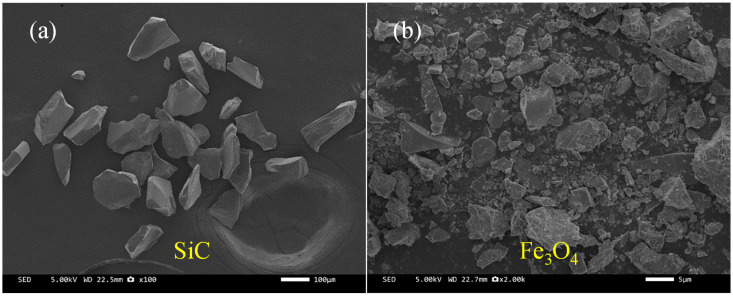
Microstructure of (**a**) SiC and (**b**) Fe_3_O_4._

In Figure 1a, SiC particles exhibit irregular flaky or angular morphologies, with sharp surfaces. Such characteristics contribute to the formation of a rigid skeleton within the asphalt matrix, thereby enhancing its high-temperature stability. The main performance parameters of Fe_3_O_4_ are summarized in Table 3. In Figure 1b, Fe_3_O_4_ particles are relatively small in size and irregular in shape, exhibiting good dispersibility, which facilitates their uniform distribution in the asphalt system. This uniform dispersion, combined with electromagnetic effects and interfacial interactions, improves the structural stability of the asphalt matrix. The slow-cracking cationic emulsified asphalt was used in this work, and its properties are listed in Table 4.

### 2.2. Sample Preparation

The preparation process of modified emulsified asphalt in this work includes the preparation of modified asphalt and the emulsification of modified asphalt. The formulation of the modified asphalt is given in Table 5.

The base asphalt was first heated in an oven (TX-881-3, Suzhou, China) at 165 °C until fully melted and then transferred into a thermostatic oil bath. The modifier, either SiC or Fe_3_O_4_, was added at a predetermined mass ratio. Subsequently, the shear head of a Fluke high-speed shear mixer (Yixuan GS-1 type high-speed shear, Cangzhou, China) was immersed below the asphalt surface, and the mixture was sheared at 3500 rpm for 30 min to obtain the modified asphalt, which was set aside for further use. Thereafter, the soap solution was poured into a preheated colloid mill (Juyu 130 type colloid mill, Laizhou, China), and the pre-weighed modified asphalt was slowly added while being stirred with a glass rod to ensure adequate mixing with the soap solution. After colloid milling for 5 min, the modified emulsified asphalt samples were obtained [21,22]. During shearing, the asphalt temperature was maintained at 165 ± 5 °C to avoid excessive viscosity that may hinder dispersion; the colloid mill gap was set to 0.1 mm for secondary refinement. The emulsified asphalt without any modifier was used as the control group.

### 2.3. Characterizations

#### 2.3.1. Electromagnetic Properties

The electromagnetic properties of the emulsified asphalt residues were tested using a vector network analyzer (Agilent E5071C, Agilent Technologies Inc., Santa Clara, CA, USA). The residues were placed into silicone molds and fabricated into rectangular specimens with dimensions of 22.86 mm in length, 10.16 mm in width, and 2 mm in thickness. The sample size is consistent with the internal cavity size of the waveguide test fixture connected to the vector network analyzer (Agilent E5071C, Agilent Technologies Inc., Santa Clara, CA, USA), ensuring the uniform propagation of electromagnetic waves in the sample and improving the accuracy of electromagnetic property testing. A vector network analyzer (E8362B, VNA, Agilent, Palo Alto, CA, USA) was then employed to measure the complex permittivity and permeability of the samples within the frequency range of 8.2–12.4 GHz using the waveguide method [23]. Each group was prepared with 3 parallel samples, and the averaged test results were adopted.

#### 2.3.2. Rheological Properties

The rheological properties of the emulsified asphalt samples were evaluated using a dynamic shear rheometer (DSR, TA DHR-1, TA Instruments, New Castle, DE, USA). The evaporation residues of the emulsified asphalt were molded into circular specimens with a diameter of 25 mm and a thickness of 1 mm. A temperature sweep test was conducted in the range of 28–76 °C using the DSR, during which the variations in complex modulus (G*) and phase angle (*δ*) were recorded to assess the effects of SiC and Fe_3_O_4_ on the viscoelastic behavior of the emulsified asphalt.

Additionally, the multiple stress creep and recovery (MSCR) test was conducted to evaluate high-temperature anti-deformation and elastic recovery. The test was performed with two stresses (0.1 kPa, 3.2 kPa), yielding key indicators: recovery percentage (*R*) and non-recoverable creep compliance (*J_nr_*) [24]. Each group was prepared with 3 parallel samples, and the averaged test results were adopted.

#### 2.3.3. Microwave Heating Property

A total of 50 g of modified emulsified asphalt was accurately weighed and evenly placed into a crucible, which was then subjected to microwave heating. During the heating process, an infrared thermal imager (Fluke TiS75, Fluke Corporation, Everett, WA, USA) was used to capture thermal images of the sample in the crucible at 20 s intervals, with the entire heating process lasting for 2 min [25]. Each group of samples was tested three times, and the average of the three measurements was taken as the final result.

#### 2.3.4. Microstructural Evolution Analysis

Different emulsified asphalt samples were drawn using a dropper and placed dropwise onto glass slides, where they were evenly spread and then covered with cover slips. The prepared slides were transferred into a microwave heating device for heating. During the heating process, the slides were removed every 20 s and immediately observed under a microscope (ISH500, Shanghai Puhe Optoelectronics Technology Co., Ltd., Shanghai, China) to examine the morphological changes of the emulsified asphalt. Each group was prepared with 3 parallel samples, and the averaged test results were adopted.

## 3. Results and Discussion

### 3.1. Dielectric Properties of Modified Emulsified Asphalt Samples

#### 3.1.1. Emulsified Asphalt Samples Modified with SiC

**Figure 2:**
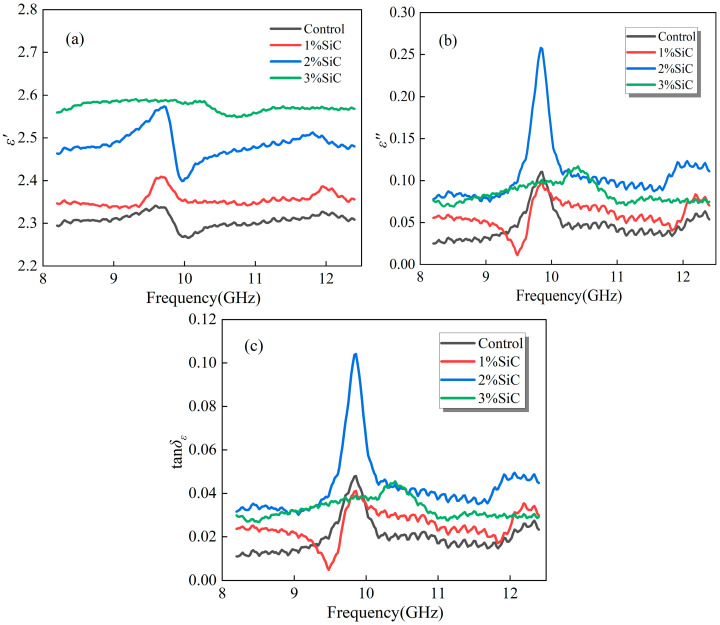
Dielectric properties of emulsified asphalt samples modified with SiC: (**a**) real part of complex permittivity (*ε*′); (**b**) imaginary part of complex permittivity (*ε*″); and (**c**) dielectric loss tangent (*tanδ_ε_*).

Figure 2 illustrates the dielectric response characteristics of emulsified asphalt samples modified with varying amounts of SiC in the 8.2–12.4 GHz frequency range. In Figure 2a, the real part of complex permittivity ε′ of the samples shows a general upward trend, which becomes particularly prominent at higher concentrations, as the SiC content increases. This indicates that the introduction of SiC effectively enhances the polarization capacity of the matrix, and its high dielectric constant contributes to the formation of more energy storage units, thereby improving the dielectric energy storage performance of the material under an electric field [26]. In contrast, the ε′ increase in the samples with lower SiC content is relatively modest, although the dielectric constant shows some improvement.

Dielectric loss (*ε*″, imaginary part of complex permittivity) represents the ability of a material to convert electromagnetic energy into thermal energy through polarization relaxation, conductive loss, and other mechanisms, during the process of storing electromagnetic energy. Loss tangent (*tanδ_ε_*) reflects the relative magnitude of dielectric loss in the material, i.e., the efficiency of converting stored electromagnetic energy into thermal energy, as shown in Equation (1).

(1) $$tan\delta_{\varepsilon }=\frac{\varepsilon^{\prime \prime }}{\varepsilon^{\prime }}$$

In terms of the imaginary part of complex permittivity (*ε*″) and dielectric loss tangent (*tanδ_ε_*), Figure 2b,c show that the sample with 2% SiC content exhibits superior energy dissipation capability. It indicates that more effective polarization relaxation and conductive loss mechanisms are established within the material, enabling the external electromagnetic energy to be converted into heat more efficiently. Although the sample still maintains a relatively high loss level when the SiC content is further increased, interparticle interactions partially weaken the energy dissipation effect, resulting in inferior performance compared with samples with moderate SiC content [27]. Overall, SiC contributes to improving the electromagnetic response of the material, while a moderate content (2%) proves to be more effective in enhancing dielectric loss.

#### 3.1.2. Emulsified Asphalt Samples Modified with Fe_3_O_4_

**Figure 3:**
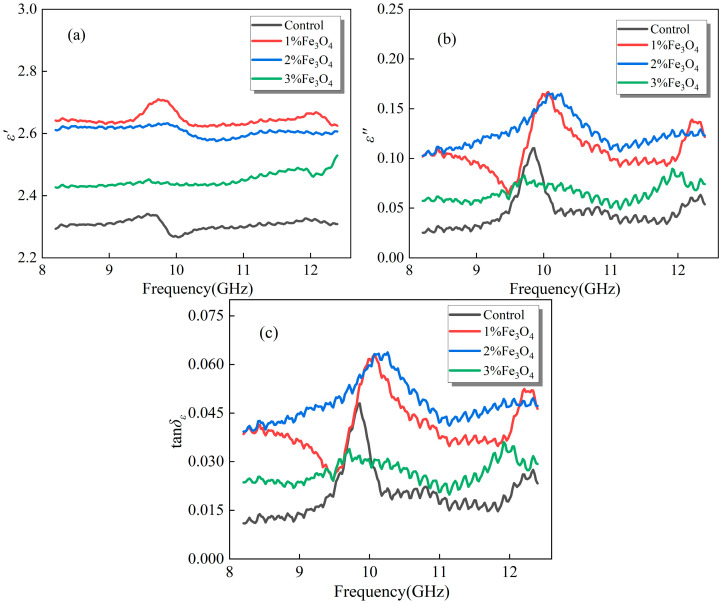
Dielectric properties of emulsified asphalt samples modified with Fe_3_O_4_: (**a**) real part of complex permittivity (*ε*′); (**b**) imaginary part of complex permittivity (*ε*″); and (**c**) dielectric loss tangent (*tanδ_ε_*).

Figure 3 presents the dielectric properties of emulsified asphalt samples modified with different Fe_3_O_4_ contents. Overall, the *ε*′ of the samples increases significantly compared to the control group with the introduction of Fe_3_O_4_. This indicates that Fe_3_O_4_ particles can introduce additional polarization interfaces within the matrix, and their inherent conductivity and magnetic characteristics further enhance the electromagnetic response of the system, enabling the material to store and transfer more electromagnetic energy under an applied electric field [28,29].

In terms of *ε*″ and *tanδ_ε_*, the addition of Fe_3_O_4_ also shows a positive effect, with the most significant improvement observed at moderate concentrations. This suggests that the synergistic action between dipolar polarization and conductive loss mechanisms within the material is most effective under these conditions, allowing electromagnetic energy to be converted into heat more efficiently. Although the loss levels of the samples at higher concentrations remain higher than those of the control group, excessive filler may lead to an increase in interface defects, which in turn partially suppresses the further enhancement of loss capabilities [30]. Therefore, it is evident that Fe_3_O_4_ modification not only enhances the dielectric energy storage performance of emulsified asphalt but also demonstrates excellent potential for energy dissipation, with the enhancement effect being highly sensitive to the filler content.

#### 3.1.3. Emulsified Asphalt Samples Co-Modified with SiC and Fe_3_O_4_

**Figure 4:**
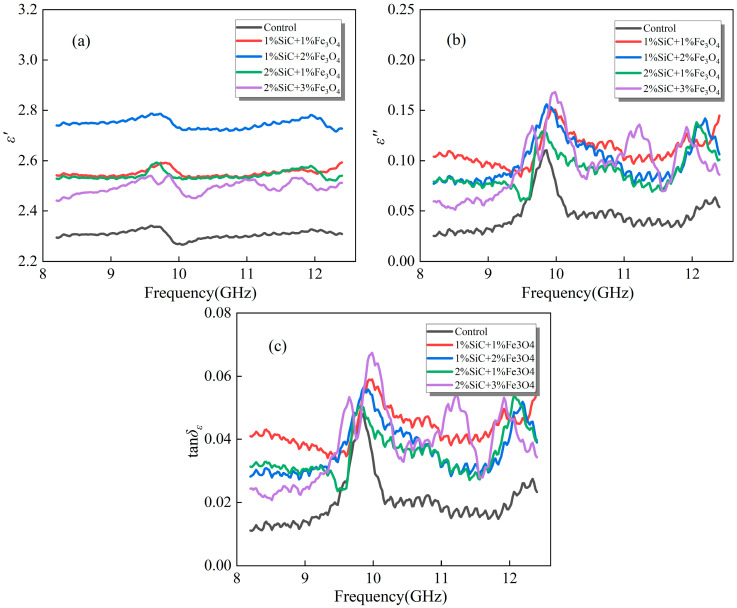
Dielectric properties of emulsified asphalt samples co-modified with SiC and Fe_3_O_4_: (**a**) real part of complex permittivity (*ε*′); (**b**) imaginary part of complex permittivity (*ε*″); and (**c**) dielectric loss tangent (*tanδ_ε_*).

Figure 4 illustrates the dielectric response of emulsified asphalt samples co-modified with SiC and Fe_3_O_4_ within the 8.2–12.4 GHz frequency range. It can be observed that the co-modified system exhibits a more pronounced increase in *ε*′ compared with the single-filler samples, indicating that the synergistic effect of the two modifiers effectively facilitates the polarization process within the matrix.

In terms of *ε*″ and *tanδ_ε_*, the co-modified samples generally outperform those with a single filler. This indicates that polarization relaxation, conductive loss, and magneto–electric coupling effects act synergistically under co-modification conditions, enabling more efficient absorption and conversion of external electromagnetic energy [31]. Notably, both *ε*″ and *tanδ_ε_* of the sample with 2% SiC + 3% Fe_3_O_4_ show significant enhancement and reach the highest values among all co-modification ratios, although its *ε*′ is relatively low. This phenomenon is primarily attributed to the magnetic loss effect induced by the higher Fe_3_O_4_ content, where the enhancement of eddy current loss and magnetic resonance effects leads to a marked increase in energy dissipation within the electromagnetic field [31]. The contribution of SiC is not fully realized at this composition, although it provides additional polarization interfaces, resulting in a relatively modest improvement in the dielectric constant, while Fe_3_O_4_ plays a dominant role in governing the overall energy dissipation process.

Moreover, local field distortion may occur within the system when the proportion of one component becomes excessively high, thereby reducing the extent of improvement in loss performance [32]. Therefore, co-modification is not a simple additive effect but rather a process of optimizing the ratio and dispersion state to achieve optimistic dielectric properties. Although the increase in dielectric constant of the sample with 2% SiC + 3% Fe_3_O_4_ is limited, the pronounced magnetic loss characteristics result in significantly improved loss performance. This suggests that this ratio is particularly suitable for applications requiring high energy dissipation.

### 3.2. Magnetic Properties of Modified Emulsified Asphalt Samples

#### 3.2.1. Emulsified Asphalt Samples Modified with SiC

**Figure 5:**
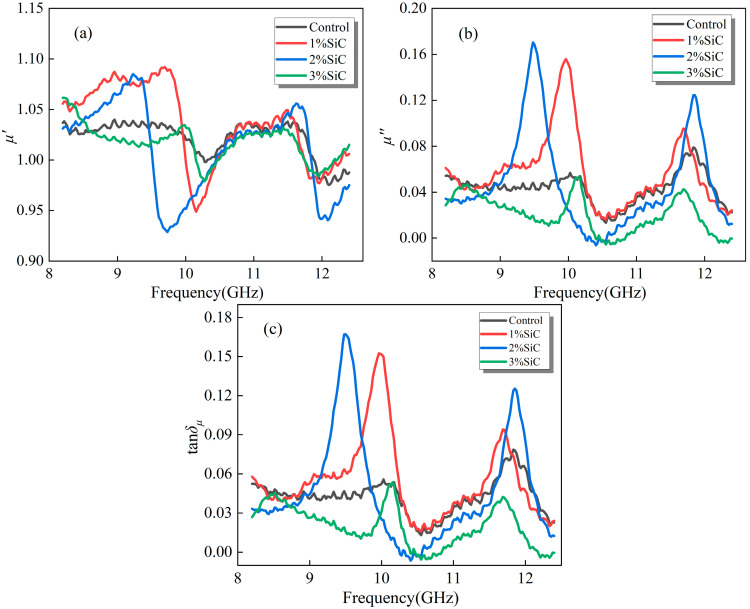
Magnetic loss properties of modified emulsified asphalt samples with SiC: (**a**) real part of complex permeability (*μ*′); (**b**) imaginary part of complex permeability (*μ*″); and (**c**) magnetic loss tangent (*tanδ_μ_*).

Figure 5 illustrates the magnetic properties of emulsified asphalt samples modified with varying silicon carbide contents in the 8.2–12.4 GHz frequency range. In Figure 5a, *μ*′ for all samples remains close to 1, with only minor frequency-dependent variations and slight local fluctuations. This confirms that silicon carbide, as a nonmagnetic ceramic filler, does not significantly alter the system’s magnetic energy storage capacity, and the material overall retains quasi-nonmagnetic characteristics.

In contrast, specimens with partial SiC doping show more distinct peaks in the *μ*″ (imaginary part of complex permeability) and *tanδ_μ_* (magnetic loss tangent, shown in Equation (2)) curves, particularly in the mid-to-high frequency range (around 9–11 GHz), compared to the control group. Although SiC itself is nonmagnetic, its high dielectric constant and electrical conductivity modify the electromagnetic field distribution of the asphalt matrix, inducing additional eddy current losses or resonance-like effects in specific frequency regions. This results in enhanced *μ*″ and *tanδ_μ_* values [33]. Moreover, the difference between low and high SiC contents is substantial, suggesting that the dispersion state and local aggregation of SiC exert a notable modulation effect on the magnetic response.

(2) $$tan\delta_{\mu }=\frac{\mu^{\prime \prime }}{\mu^{\prime }}$$

#### 3.2.2. Emulsified Asphalt Samples Modified with Fe_3_O_4_

**Figure 6:**
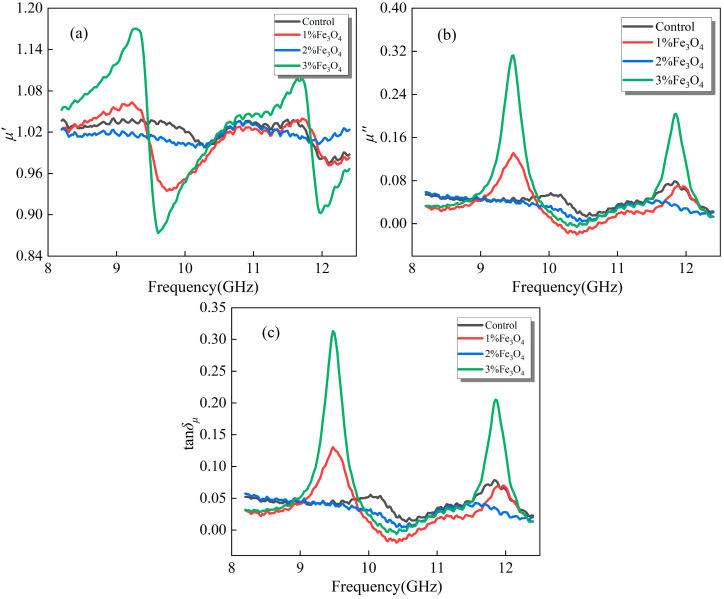
Magnetic loss properties of modified emulsified asphalt samples with Fe_3_O_4_: (**a**) real part of complex permeability (*μ*′); (**b**) imaginary part of complex permeability (*μ*″); and (**c**) magnetic loss tangent (*tanδ_μ_*).

Figure 6 illustrates the magnetic permeability variation of emulsified asphalt samples modified with different Fe_3_O_4_ dosages in the 8.2–12.4 GHz frequency range. From the *μ*′ curves, increasing Fe_3_O_4_ content leads to pronounced undulating fluctuations in certain frequency intervals, with larger deviations observed at higher doping levels. This demonstrates that Fe_3_O_4_ effectively alters the magnetic energy storage capacity of the material and produces an enhanced magnetic response at specific frequencies.

In the *μ*″ and *tanδ_μ_* curves, distinct peaks appear in all Fe_3_O_4_-doped samples, and peak intensity increases significantly with higher doping, with the heavily doped group showing the most prominent magnetic loss peaks near 9–11 GHz. This behavior primarily arises from the ferromagnetic and semiconducting nature of Fe_3_O_4_, whose particles undergo natural resonance and vortex effects under an applied electromagnetic field, thereby markedly enhancing system energy dissipation within certain frequency intervals [19]. Compared with the control and low-doped samples, high Fe_3_O_4_ loading not only raises the *μ*″ peak but also yields higher *tanδ_μ_*, indicating that magnetic loss mechanisms dominate the system’s electromagnetic response. Furthermore, Fe_3_O_4_ incorporation introduces an additional loss channel beyond dielectric loss, significantly strengthening the electromagnetic response and improving electromagnetic energy conversion efficiency [3].

#### 3.2.3. Emulsified Asphalt Samples Co-Modified with SiC and Fe_3_O_4_

**Figure 7:**
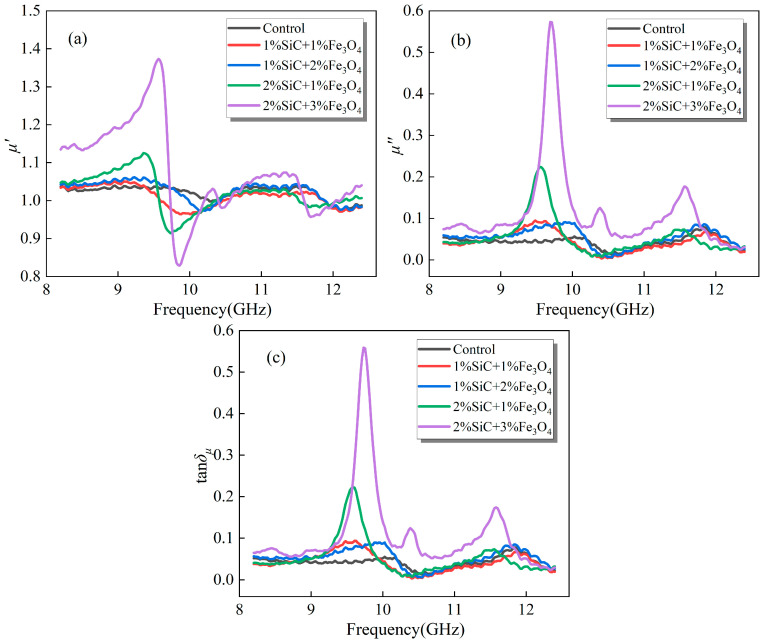
Magnetic loss properties of modified emulsified asphalt samples compounded with SiC and Fe_3_O_4_: (**a**) real part of complex permeability (*μ*′); (**b**) imaginary part of complex permeability (*μ*″); and (**c**) magnetic loss tangent (*tanδ_μ_*).

Figure 7 shows that the overall *μ*′ of the complex-doped system remains close to 1 within the 8.2–12.4 GHz band. However, the 2% SiC + 3% Fe_3_O_4_ group exhibits strong dispersion near 9–10 GHz, characterized by a steep rise in μ′, followed by a rapid drop, briefly falling below 1 before gradually stabilizing. In synchrony, *μ*″ and *tanδ_μ_* display sharp peaks in the same frequency range. This behavior can be attributed to the pronounced magnetic resonance of Fe_3_O_4_, while SiC enhances substrate conductivity and dielectric properties, thereby strengthening localized circulating currents and magnetic induction intensity and amplifying the magnetic loss channel [20].

The SiC-Fe_3_O_4_ composite introduces dual electrical and magnetic loss pathways. SiC amplifies eddy currents and interfacial relaxation through conductive and dielectric regulation, whereas Fe_3_O_4_ contributes strong magnetic resonance and hysteresis dissipation. When their ratio and dispersion state are appropriately matched, synergistic enhancement of *μ*″ and *tanδ_μ_* can be achieved, together with bandwidth expansion. Consequently, the system achieves synergistic optimization of microwave absorption and thermal conversion performance [29].

### 3.3. Microwave Heating Property of Modified Emulsified Asphalt Samples

**Figure 8:**
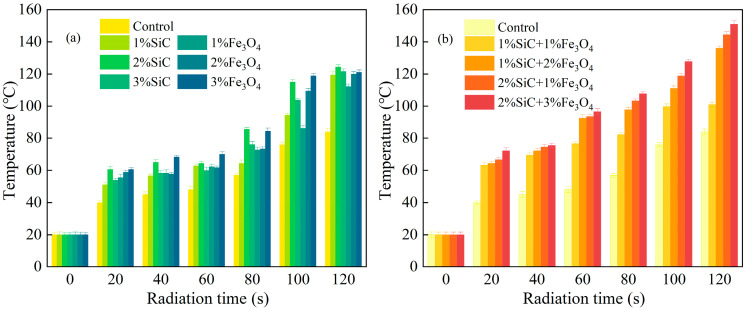
Microwave heating effect of modified emulsified asphalt samples: (**a**) single-doped composites; and (**b**) compound-doped composites.

Figure 8 presents the heating curves of modified emulsified asphalt under identical microwave power with different filler ratios. Overall, both the heating rates and final temperatures of all filler systems exceed those of the control group, confirming that electromagnetic fillers markedly improve microwave energy absorption and conversion within the material.

For the single-doped systems, 3% Fe_3_O_4_ increases the early heating rate (0–60 s) by about 102.7% compared with the control, reflecting the typical rapid warming dominated by magnetic losses. In contrast, 2% SiC yields an end-temperature rise of 41.7% and a rate increase of 64.2%, showing dielectric-dominated behavior characterized by a high ε′ with moderate loss. Since SiC conductivity rises with temperature, this effect may stem from the temperature-induced refinement of conductive networks and accelerated polarization relaxation, which rapidly amplify dielectric-conductive losses in the mid-to-late stage, ultimately producing a high warming rate.

The compound-doped systems exhibit stronger early heating capability. For instance, the linear heating rate of 2% SiC + 3% Fe_3_O_4_ in 0–60 s increases by about 176.2% compared with the control, while 2% SiC + 1% Fe_3_O_4_ and 1% SiC + 2% Fe_3_O_4_ also reach 161.8% and 159.8%, respectively. These results align with the enhanced dielectric loss (*ε*″, *tanδ*) and magnetic loss (*μ*″) of the compound-doped samples, as well as the broadening of *μ*″ in the mid-to-high frequency range, indicating that electrical and magnetic loss channels are synergistically activated, leading to higher initial absorption and a faster temperature rise.

Overall, compound doping is more effective than single doping in achieving simultaneous optimization of energy storage and loss, thereby improving both early heating rate and final temperature. However, compound doping is not a simple additive effect. At high component ratios, local field distortion may weaken polarization and relaxation efficiency, resulting in mismatches between *ε*′ and *ε*″. Based on the above analysis, a loss-oriented synergistic ratio is recommended for applications prioritizing rapid heating and high-efficiency energy conversion. Conversely, when stability of temperature rise and energy storage is emphasized, formulations with a higher dielectric plateau and moderate loss should be preferred.

### 3.4. Rheological Properties of Modified Emulsified Asphalt Samples

#### 3.4.1. Complex Modulus, Phase Angle, and Rutting Factor Analyses

**Figure 9:**
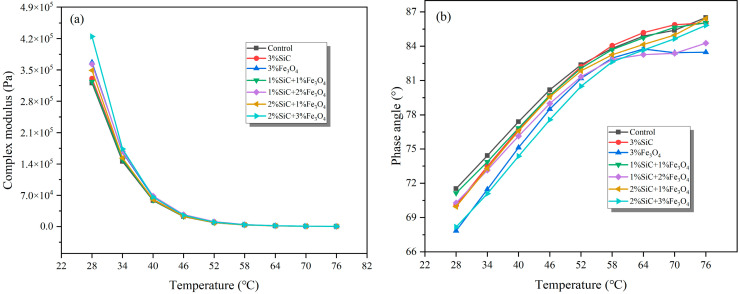
Rheological properties of modified emulsified asphalt evaporation residue samples: (**a**) complex modulus; and (**b**) phase angle.

The results of the temperature scanning test are shown in Figure 9. The complex modulus significantly decreases with increasing temperature, indicating that the stiffness of emulsified asphalt residue decreases with increasing temperature. However, the complex modulus is increased with the increase in modifier content, reflecting the improvement in material mechanical properties. Among all the modified groups, the improvement effect of Fe_3_O_4_ on the rheological properties of emulsified asphalt evaporation residue is more significant than that of SiC, showing a higher complex modulus, indicating that Fe_3_O_4_ can more effectively enhance the high-temperature stiffness of emulsified asphalt evaporation residue.

The trend in phase angle variation is opposite to that of complex modulus and is clearly controlled by the type and dosage of modified materials. All modified groups showed lower phase angles, especially the 2% SiC + 3% Fe_3_O_4_ group samples, whose phase angle decrease is significantly greater than that of other modified groups. This indicates that the group exhibits strong elastic recovery ability and can significantly improve the rheological stability of emulsified asphalt evaporation residue.

**Figure 10:**
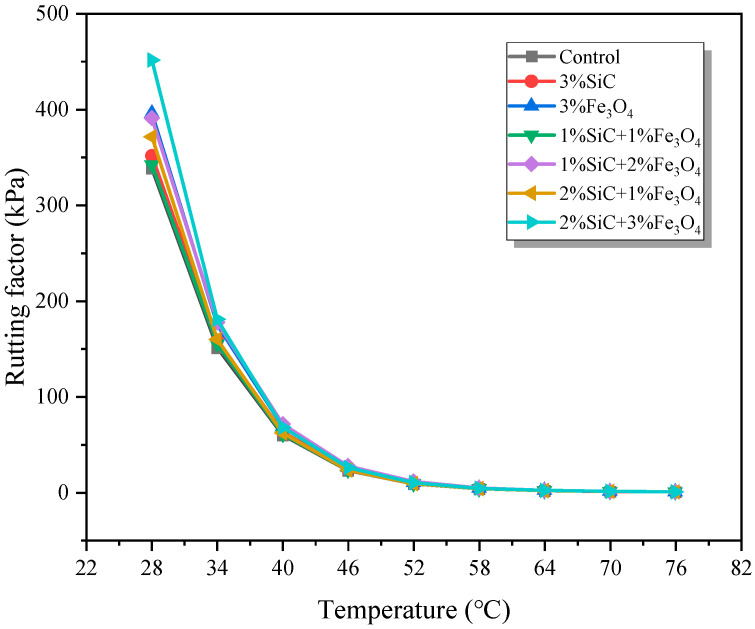
Rutting factor of modified emulsified asphalt evaporation residue samples.

The calculation results of the rutting factor are shown in Figure 10. The rutting factor of all group samples gradually decreases with increasing temperature, but the modified group samples exhibit a higher rutting factor at the same temperature. The rutting factor of the control group is at the lowest level among all sample groups, indicating that its resistance to rutting is the worst. The rutting factor of the 3% SiC- and 3% Fe_3_O_4_-modified emulsified asphalt samples remained at a higher level compared to the control group, especially in the medium-to-high temperature range, showing strong high-temperature stability and anti-rutting ability. The performance of the compounded system is more prominent, indicating that the synergistic effect of SiC and Fe_3_O_4_ enhances the high-temperature stiffness of the material while also improving its elastic recovery ability. Overall, the rutting factor of modified asphalt exhibits stronger high-temperature stability with the increase in Fe_3_O_4_ content, indicating that the addition of Fe_3_O_4_ has a more significant improvement in high-temperature rutting resistance.

#### 3.4.2. Creep and Recovery Analyses

**Figure 11:**
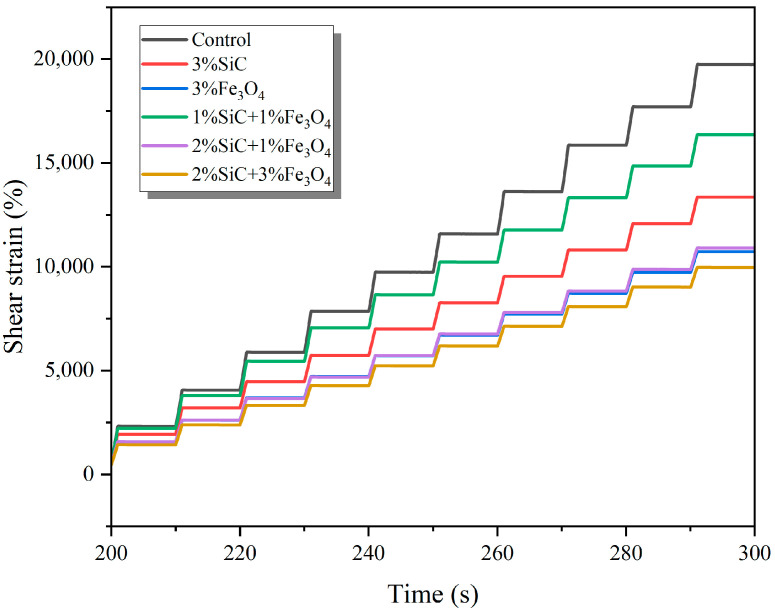
Typical strain–time curve of modified emulsified asphalt evaporation residue samples (3.2 kPa, 200–300 s).

The MSCR test results are shown in Figure 11. It can be observed that the control group accumulates shear strain the fastest during the loading process and exhibits poor resistance to external forces. In addition, the shear strain of all modified groups is significantly lower than that of the control group, indicating that both the single-mixed and compounded system can significantly improve the anti-deformation ability of emulsified asphalt evaporation residue. Among them, the addition of SiC mainly slows down the creep rate by increasing the stiffness of asphalt, while the effect of Fe_3_O_4_ is more significant, not only reducing creep strain but also showing stronger recovery ability during the unloading stage [19]. The compounded system balances the improvement in stiffness and recovery, exhibiting better anti-rutting potential than the single-mixed system.

**Figure 12:**
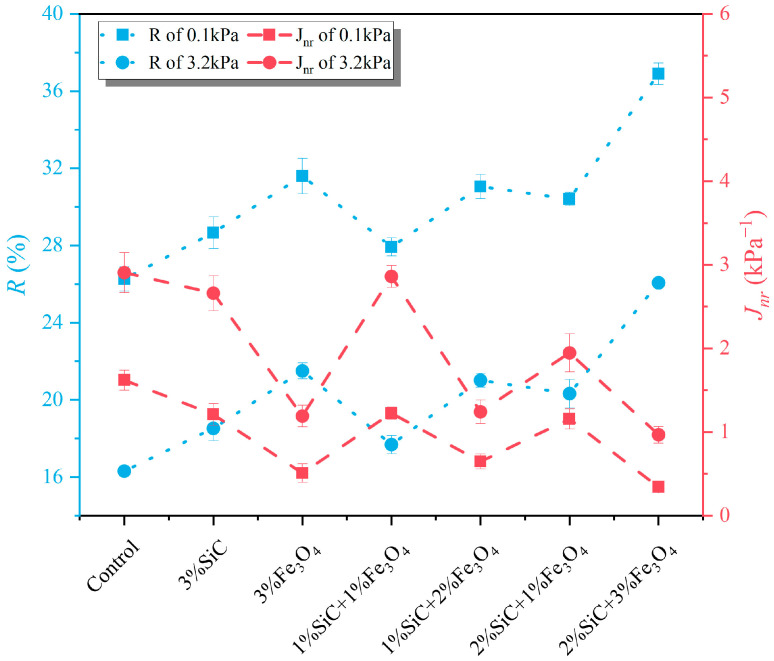
Recovery percentage *R* and non-recoverable creep compliance *J_nr_* of modified emulsified asphalt evaporation residue samples under different stress levels.

The recovery rate (*R*) and unrecoverable creep compliance (*J_nr_*) of different modified samples are shown in Figure 12. It can be seen that the R values of all modified groups are higher than those of the control group, while the *J_nr_* value is significantly reduced, further indicating that the modified system can improve the elasticity of emulsified asphalt evaporation residue and reduce permanent deformation. Among the modifications, the Fe_3_O_4-_modified system exhibits particularly outstanding effects in improving *R* and reducing J_nr_, outperforming the SiC-modified group. This indicates that Fe_3_O_4_ contributes more to improving the rheological properties of asphalt. The composite system with SiC and Fe_3_O_4_ exhibits higher R and lower J_nr_ at different stress levels. The R (3.2 kPa) of the 2% SiC + 3% Fe_3_O_4_ group sample increased by 59.7%, and the *J_nr_* (3.2 kPa) decreased by 68.9% compared to the control group. This indicates that the synergistic effect of SiC and Fe_3_O_4_ can effectively improve the comprehensive rheological properties of asphalt at high temperatures.

### 3.5. Morphological Changes During Microwave Radiation

**Figure 13:**
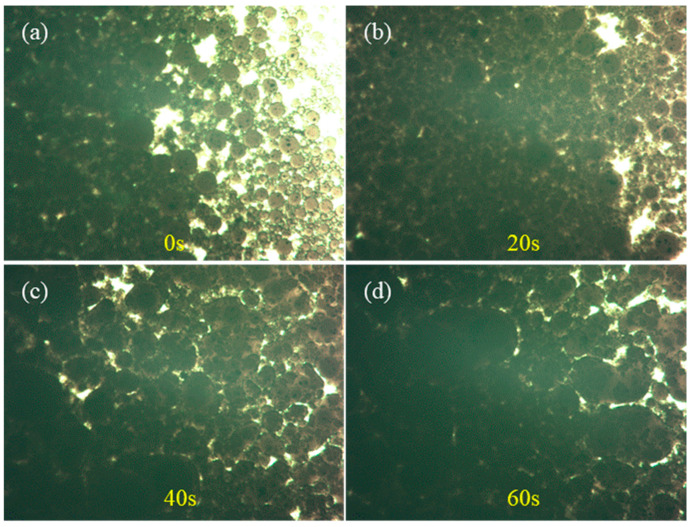
Particle morphology changes of 1% SiC + 1% Fe_3_O_4_ group samples under microwave radiation for (**a**) 0 s; (**b**) 20 s; (**c**) 40 s; and (**d**) 60 s.

The morphological evolution of emulsified asphalt particles modified by 1% SiC + 1% Fe_3_O_4_ composite during 1 min of microwave radiation is shown in Figure 13. In the initial stage (0 s, Figure 13a), the particle distribution is relatively dense, and the interface is clear, presenting a uniformly dispersed state. As the radiation time extended to 20 s (Figure 13b), the boundaries between particles gradually became blurry, and local melting and bonding phenomena gradually occurred, indicating that fusion driven by thermal effects began to occur inside the system. At 40 s (Figure 13c), the particle gaps were further reduced, presenting a more continuous structure, with some areas forming a clear network of connections. This indicates that SiC and Fe_3_O_4_ promote the flow and interaction of the asphalt matrix under microwave action. By 60 s (Figure 13d), the particles tend to be evenly distributed, and the interface transition becomes smoother, indicating that the material gradually undergoes a transition from a granular to a continuous phase under microwave radiation.

This evolutionary trend indicates that the demulsification process of emulsified asphalt can be significantly accelerated by microwave radiation. This may be due to the fact that SiC can provide skeletal support in the composite structure, while Fe_3_O_4_ enhances energy absorption and local heating through electromagnetic effects. Therefore, the emulsification and blending rate between emulsified asphalt particles are enhanced through the synergistic effect of the two materials.

## 4. Conclusions

Microwave-assisted pavement repair technology is crucial for improving the efficiency of road maintenance and reducing social costs, but the limited microwave heating performance of conventional emulsified asphalt restricts its application. This work aimed to address this issue by preparing microwave-sensitive emulsified asphalt using SiC and Fe_3_O_4_ as composite modifiers. In this work, the electromagnetic properties, microwave heating properties, rheological properties, and microstructural evolution law of microwave-sensitive emulsified asphalt modified by single or multiple doping of SiC and Fe_3_O_4_ were investigated, and the main conclusions are as follows:

(1) The SiC-Fe_3_O_4_ sample synergistically enhances the electromagnetic properties of the emulsified asphalt, with a more significant increase in dielectric constant and higher loss peaks compared to the single-filler systems. Among the samples, 1% SiC + 2% Fe_3_O_4_ (mass fraction; same below) achieves a balance between dielectric energy storage and dissipation, while 2% SiC + 3% Fe_3_O_4_ exhibits the optimal energy dissipation capability.
(2) The two modifiers can effectively enhance the microwave heating performance of emulsified asphalt, with the co-modified system outperforming the single-filler system. The co-modification of SiC and Fe_3_O_4_ shows a greater increase in early heating rate and a significantly higher final temperature compared to the control sample, with synergistic improvements in both electrical and magnetic loss properties.
(3) The co-modification of SiC and Fe_3_O_4_ synergistically optimizes the rheological properties of the emulsified asphalt, with a more significant increase in the rutting factor and improved elastic recovery compared to the single-filler systems. The 2% SiC + 3% Fe_3_O_4_ sample exhibits the largest decrease in phase angle, and Fe_3_O_4_-SiC balances both stiffness and elastic recovery in the intermediate-to-high temperature range.
(4) Microwave radiation accelerates the demulsification and fusion process of the co-modified emulsified asphalt. The sample with 1% SiC + 1% Fe_3_O_4_ transitions from a densely dispersed particle state to a continuous phase with reduced gaps within 0–60 s of heating, ultimately forming a smooth and uniform structure at the interface.
(5) The co-modification of SiC and Fe_3_O_4_ achieves a synergistic enhancement of the electromagnetic properties, microwave heating efficiency, and high-temperature rheological stability of emulsified asphalt. Among the samples, 1% SiC + 2% Fe_3_O_4_ is suitable for applications that emphasize overall stability, while 2% SiC + 3% Fe_3_O_4_ is ideal for engineering scenarios that require efficient heating.

## 5. Further Research

The particle size of modifiers, their chemical stability under microwave cycles, and their heating–cooling cycle behavior can influence the long-term service performance of SiC-Fe_3_O_4_-modified microwave-sensitive emulsified asphalt. Proper exploration of these factors can be beneficial for balancing its electromagnetic, heating, and rheological properties, with positive effects on engineering applicability. However, insufficient investigation of these aspects may result in unclear guidance for large-scale promotion. Therefore, further research is necessary to investigate the influence of these factors on the material’s long-term performance.

## Figures and Tables

**Table 1:** Chemical compositions of SiC.

Contents (%)	Fe_2_O_3_ (%)	Free Carbon (%)	Molecular Weight (g/mol)
≥98.5	≤0.5	≤0.25	40.1

**Table 2:** Physical properties of SiC.

Mohs Hardness	Loss on Ignition (%)	Water Content (%)	Melting Point (°C)
9.3	≤0.2	≤0.3	2700 °C

**Table 3:** Performance parameters of Fe_3_O_4._

Mohs Hardness	Apparent Density (g/cm^2^)	Loss on Ignition (%)	Water Content (%)
5.5	1.356	0.5	0.5

**Table 4:** Properties of emulsified asphalt.

Properties		Results
The remaining amount on the sieve (1.18 mm)/%		0.05
Storage stability (1 d, 25 °C)/%		0.7
Storage stability (5 d, 25 °C/%		2.5
Evaporation residue	Evaporation residue content/%	62.3
	Penetration (25 °C, 100 g, 5 s)/0.1 mm	73
	Softening point/°C	48.2
	Ductility (25 °C)/cm	125

**Table 5:** Mix proportion of samples.

Samples	SiC	Fe_3_O_4_	Total Additive Amount (Mass Fraction)
Control	0%	0%	0%
1% SiC	1%	0%	1%
2% SiC	2%	0%	2%
3% SiC	3%	0%	3%
1% Fe_3_O_4_	0%	1%	1%
2% Fe_3_O_4_	0%	2%	2%
3% Fe_3_O_4_	0%	3%	3%
1% SiC + 1% Fe_3_O_4_	1%	1%	2%
1% SiC + 2% Fe_3_O_4_	1%	2%	3%
2% SiC + 1% Fe_3_O_4_	2%	1%	3%
2% SiC + 3% Fe_3_O_4_	2%	3%	5%

## Data Availability

The original contributions presented in this study are included in the article. Further inquiries can be directed to the corresponding author(s).
